# Exploring the Contribution of Estrogen to Amyloid-Beta Regulation: A Novel Multifactorial Computational Modeling Approach

**DOI:** 10.3389/fphar.2013.00016

**Published:** 2013-03-01

**Authors:** Thomas J. Anastasio

**Affiliations:** ^1^Computational Neurobiology Laboratory, Beckman Institute, Department of Molecular and Integrative Physiology, University of Illinois at Urbana-ChampaignUrbana, IL, USA

**Keywords:** Alzheimer disease, amyloid-β, estrogen, computational model, declarative programming, formal methods, multifactorial process, multi-drug therapy

## Abstract

According to the amyloid hypothesis, Alzheimer Disease results from the accumulation beyond normative levels of the peptide amyloid-β (Aβ). Perhaps because of its pathological potential, Aβ and the enzymes that produce it are heavily regulated by the molecular interactions occurring within cells, including neurons. This regulation involves a highly complex system of intertwined normative and pathological processes, and the sex hormone estrogen contributes to it by influencing the Aβ-regulation system at many different points. Owing to its high complexity, Aβ regulation and the contribution of estrogen are very difficult to reason about. This report describes a computational model of the contribution of estrogen to Aβ regulation that provides new insights and generates experimentally testable and therapeutically relevant predictions. The computational model is written in the declarative programming language known as Maude, which allows not only simulation but also analysis of the system using temporal-logic. The model illustrates how the various effects of estrogen could work together to reduce Aβ levels, or prevent them from rising, in the presence of pathological triggers. The model predicts that estrogen itself should be more effective in reducing Aβ than agonists of estrogen receptor α (ERα), and that agonists of ERβ should be ineffective. The model shows how estrogen itself could dramatically reduce Aβ, and predicts that non-steroidal anti-inflammatory drugs should provide a small additional benefit. It also predicts that certain compounds, but not others, could augment the reduction in Aβ due to estrogen. The model is intended as a starting point for a computational/experimental interaction in which model predictions are tested experimentally, the results are used to confirm, correct, and expand the model, new predictions are generated, and the process continues, producing a model of ever increasing explanatory power and predictive value.

## Introduction

It is by now well known that the Women’s Health Initiative found no benefit of estrogen therapy on cognitive function for post-menopausal women (for reviews, see Anderson et al., 2004; Espeland et al., 2004). However, a recent evidence-based statement indicates that estrogen therapy may be effective, but it must be initiated not after menopause but during the perimenopausal period (North American Menopause, 2012). The lack of unequivocal benefit of estrogen therapy on cognitive function is surprising, considering that estrogen receptors α and β (ERα and ERβ) are found throughout the hippocampus and cortex, the two brain regions most implicated in Alzheimer Disease (AD; Shughrue and Merchenthaler, 2000), and that estrogen is effective in lowering brain amyloid-β (Aβ) levels in ovariectomized transgenic mice that overexpress Aβ (e.g., Levin-Allerhand et al., 2002; Carroll et al., 2007). Many other factors are also involved in the pathogenesis of AD, and it is possible that the failure to take them into account has obscured the potential benefit of estrogen therapy. The purpose of this computational modeling study is to begin to account for the many factors in addition to estrogen that participate in the regulation of Aβ, and to explore ways in which estrogen therapy might be used more effectively in AD treatment, perhaps by administering estrogen in conjunction with other agents. The modeling results can be considered as predictions concerning the effects of administration of estrogen, ER agonists, and other agents, alone or in combination, on the level of Aβ. Verification of these predictions in mouse models of AD would suggest potential new avenues for the development of pharmacological strategies for AD treatment.

It has been recognized for over a decade that neurodegenerative diseases such as AD are highly complex and multifactorial (e.g., Jellinger, 2003). Meanwhile, the database of experimental findings on AD grows at an accelerating pace. The sheer size and complexity of the AD database poses a barrier to understanding this disease. Computational methods are needed to represent the many interactions that are involved in AD and to simulate and analyze them in order to gain insight into the disorder, and to generate experimentally testable hypotheses that are also relevant to possible treatments. Recently, powerful computational methods for simulating and analyzing complex systems have been imported from computer science into biology (for reviews, see Hlavacek et al., 2006; Fisher and Henzinger, 2007). These methods, known as formal methods in computer science, are implemented using declarative programming languages.

Computationally modeling a system using declarative programming permits not only simulation but also analysis of the system being modeled (Huth and Ryan, 2004). The analysis capability is essential for reasoning about complex neurodegenerative disorders such as AD. The declarative programming language used for the simulations and analyses presented here is known as Maude (Clavel et al., 2007). Maude has been previously used for modeling biological systems (Eker et al., 2002; Talcott, 2008), and Maude has been recently used to model some of the molecular and cellular interactions that underlie Aβ regulation (Anastasio, 2011). That model is extended here to include many of the contributions that estrogen makes to Aβ regulation, and the estrogen-Aβ model will provide new insights into the multifactorial role of this hormone in preventing AD.

The estrogen-Aβ model will also be used to explore the possible benefits of combining other compounds with estrogen, or with ER agonists, to enhance their ability to reduce Aβ levels. In recognition of AD multifactoriality, some recent experimental studies have focused on single molecules that act as multitarget drugs (Bajda et al., 2011; Leon and Marco-Contelles, 2011). Whether the treatment involves one drug with multiple targets or multiple drugs with single targets, a consensus is emerging that preventing or treating a multifactorial disease such as AD will require a multitarget approach. The computational simulation and analysis methods presented here are ideal for generating new hypotheses on multitarget therapies for AD.

## Materials and Methods

The model is based on the “amyloid hypothesis,” according to which AD results from the buildup beyond normative levels of the Aβ peptide (Hardy and Selkoe, 2002). The model concerns the regulation of Aβ and the transition from the normative to the pathological state, rather than the state of advanced AD pathology in which Aβ levels have been chronically elevated. Although several possible triggers for Aβ buildup have been proposed, the model incorporates the subsidiary hypothesis that incipient cerebrovascular disease (CVD) can cause changes on the molecular level that dysregulate Aβ and lead to its buildup (Scheibel et al., 1989; de la Torre, 2009). The effects of estrogen on Aβ regulation are analyzed with and without the dysregulating effects of CVD. In order to focus attention on the effects of estrogen on Aβ regulation, any protection from CVD due to estrogen is disregarded.

### Biological background: Basic Aβ regulation

The Aβ peptide occurs in two lengths, Aβ_40_ and Aβ_42_, and both are toxic after they self-aggregate (Walsh and Selkoe, 2007; Di Carlo, 2010). For simplicity here, no distinction will be made between Aβ_40_ and Aβ_42_. The Aβ peptide is produced via cleavage of amyloid precursor protein (APP) by β-secretase (BACE1 or simply BACE; Vassar et al., 1999, 2009) followed by γ-secretase (Borchelt et al., 1996; De Strooper, 2003). Another enzyme, α-secretase (Esch et al., 1990; Sisodia, 1992; Vingtdeux and Marambaud, 2012) competes with BACE for APP as its substrate. Cleavage of APP by α-secretase precludes cleavage of APP by BACE, so that cleavage of APP by α-secretase precludes Aβ formation. The three secretases, α, β, and γ, and their substrate, APP, are all membrane proteins.

The γ-secretase enzyme occurs as a complex composed of presenilin-1 (PS1), presenilin enhancer-2 (PEN2), nicastrin, and anterior pharynx-defective phenotype-1 (APH1; De Strooper, 2003). APH1 is not represented in the model because little is known of its regulation, but it is assumed to be present constitutively. Regulation of the other γ-secretase components is represented in the model. Expression of PS1 and PEN2 is upregulated by c-Jun, which is phosphorylated by c-Jun N-terminal kinase (JNK) after JNK is activated by oxidative stress (OS; Tamagno et al., 2008). In contrast, the extracellular signal regulated mitogen-activated kinase (ERK) phosphorylates nicastrin and thereby downgrades its activity (Kim et al., 2006). Buildup of Aβ leads to OS and ERK activation (Bodles and Barger, 2005; Kim et al., 2006). Thus, Aβ exerts opposing influences on γ-secretase because it upregulates two of its components (PS1 and PEN2 via OS, JNK, and c-Jun) but downgrades one of its components (nicastrin via ERK; Tamagno et al., 2009).

Both normative and pathological mechanisms regulate BACE. Two normative loops operate at the RNA level. In one, Aβ upregulates expression of the BACE antisense transcript (BACEASRNA), which stabilizes the BACE message (BACEmRNA), and thereby increases BACE protein expression (Faghihi et al., 2008). In another, Aβ downregulates expression of micro-RNA-107 (BACEmiRNA), which binds to a micro-RNA recognition element on BACEmRNA and reduces its translation to BACE (Wang et al., 2008). Both of these mechanisms increase BACE protein expression, because Aβ upregulates BACEASRNA, which increases BACE expression, while Aβ downregulates BACEmiRNA, which decreases BACE expression. Note that the second loop achieves increase via decrease of decrease. In general, any pathway or loop with an even number of decreases will produce increase, just as in algebra, multiplication of an even number of negatives yields a positive.

Two pathological loops regulating Aβ are positive because they involve even numbers of negative interactions. In the first, Aβ buildup leads to activation of cytokines (Udan et al., 2008; Bulgarelli et al., 2009), which downregulate expression of the message for peroxisome proliferator-activated receptor γ (PPARmRNA), but PPAR downregulates BACEmRNA (Sastre et al., 2006). Thus, Aβ drives its own buildup by activating cytokines, which suppress PPAR, which is a suppressor of BACE, which is the enzyme most responsible for Aβ production. Non-steroidal anti-inflammatory drugs (NSAIDs) can diminish the effects on Aβ buildup of this positive feedback loop by upregulating PPAR (Sastre et al., 2006).

The second pathological positive feedback loop involves four negative interactions. Buildup of Aβ causing OS leads to release from mitochondria of second mitochondrial-derived activator of caspase (Smac; Yin et al., 2002). Smac binds to and inhibits XIAP, a member of the inhibitor of apoptosis protein (IAP) family of proteins. IAPs inhibit caspase-9. Thus, release of Smac activates caspase-9 by inhibiting IAPs, thereby releasing caspase-9 from IAP inhibition. Activation of caspase-9 leads to activation of caspase-3 leading to apoptosis (Estaquier et al., 2012). Another function of caspase-3 is to cleave, and thereby inactivate, Golgi-localized γ-ear-containing ADP-ribosylation-factor binding protein (GGA3; ADP is adenosine di-phosphate; Tesco et al., 2007). Because GGA3 participates in BACE degradation, caspase-3 activation increases BACE availability by suppressing its degradation. Thus, around a positive feedback loop involving OS and the apoptotic pathway, Aβ drives its own accumulation through two double negatives, the result of which is to reduce BACE degradation. The degradation-reducing effects of this loop can be partly diminished by seladin-1, which decreases caspase-3 activity (Sarajärvi et al., 2009), but BACE degradation can be augmented by sorting nexin 6 (SNX6; Muhammad et al., 2008; Small, 2008).

Less is known of the regulation of α-secretase than of γ- or β-secretase (BACE), but the A disintegrin and metalloprotease 10 and 17 (ADAM10 and ADAM17) family members likely perform the α-secretase function (Allinson et al., 2003). Either or both of ADAM10 and ADAM17 may be regulated by protein kinase C (PKC).

The cerebrovascular insufficiency due to CVD causes both hypoxia (reduction in oxygen level) and ischemia (reduction in blood flow), and can alter Aβ processing by both routes. The reduction in oxygen level activates hypoxia inducible factor-1-α (HIF1α or simply HIF), which upregulates BACEmRNA transcription (Zhang et al., 2007; Guglielmotto et al., 2009). The energy deprivation due to ischemia activates pancreatic endoplasmic reticulum eIF2-α kinase (PERK), which phosphorylates eukaryotic initiation factor-2-α (eIF2α or simply eIF2), which in turn de-represses a regulatory element on BACEmRNA and increases BACE protein translation (O’Connor et al., 2008). The increases in BACE protein due to CVD lead to increases in Aβ.

The low-density lipoprotein receptor-related protein (LRP) is involved in multiple but conflicting ways in Aβ processing. It increases Aβ by binding APP and then making APP more available to BACE and γ-secretase (Ulery et al., 2000; Yoon et al., 2007; Lakshmana et al., 2008). In contrast, LRP decreases Aβ by binding apolipoprotein E (apoE), whereupon the apoE, along with the Aβ it has bound, is internalized and the Aβ is degraded in the lysosome (Strittmatter et al., 1993; Manelli et al., 2004). Also, LRP decreases Aβ by binding it and transcytosing it from the brain into the peripheral circulation over the brain epithelial cells (BECs) that compose the blood-brain barrier (Shibata et al., 2000; Deane et al., 2004). In its transcytotic role LRP works with P-glycoprotein (Pgp), which transcytoses Aβ out of the brain (Cirrito et al., 2005; Kuhnke et al., 2007), but it works against the receptor for advanced glycation end products (RAGE), which transcytoses Aβ in the opposite direction, into the brain (Deane et al., 2003).

Other molecular mechanisms that regulate Aβ include reticulon-3 (RTN3) and heparan sulfate, both of which bind BACE and inhibit Aβ production (Scholefield et al., 2003; He et al., 2004; Kume et al., 2009). Receptor-associated protein (RAP) binds Aβ and causes its internalization and degradation (Kanekiyo and Bu, 2009). Enzymes that degrade Aβ include neprilysin (NEP), insulin degrading enzyme (IDE), and angiotensin-converting enzyme (ACE; Hu et al., 2001; Sudoh et al., 2002; Kanemitsu et al., 2003; Hiltunen et al., 2009). The mechanisms outlined in the subsection are included in a previous model of Aβ regulation (Anastasio, 2011). They represent many of the basic mechanisms of Aβ regulation that have been described by recent research.

### Biological background: Influences of estrogen

The main focus of the analysis described here is on the contribution of estrogen to Aβ regulation. More specifically, the model represents not fewer than nine different ways in which estrogen lowers Aβ levels. The influences of estrogen involve classical changes in protein expression levels as well as direct effects of estrogen on cell signaling. These influences are represented in the model in the context of the basic mechanisms of Aβ regulation outlined in the previous subsection. As for the basic mechanisms, the estrogen-mediated effects outlined in this subsection include many of those that have been described by recent research. Although most animal-based and *in vitro* experiments employ 17-β-estradiol rather than other forms of estrogen, the generic term estrogen will be used here for simplicity.

Estrogen binds and activates both PKCα and PKCδ (Alzamora et al., 2007). This binding and activation are direct and occur independently of estrogen binding to estrogen receptors. PKCα, but not PKCδ, shifts APP processing from BACE to α-secretase *in vitro* (Cisse et al., 2011). Such a shift from away from the amyloidogenic pathway decreases Aβ levels. These effects were due to activation of ADAM10 and/or ADAM17 by PKCα, and were not due to activation of ERK, which activates ADAM17 for functions other than APP processing.

Estrogen causes astrocytes to increase their PPAR expression *in vitro* (Valles et al., 2010), probably via upregulation of PPAR mRNA. Estrogen also increases seladin-1 mRNA and seladin-1 protein expression *in vitro* (Benvenuti et al., 2005). This seladin-1 expression reduces the activation of caspase-3 by Aβ (Luciani et al., 2008). Specific agonists of ERα were much more effective in upregulating seladin-1 than specific agonists of ERβ, demonstrating that estrogen upregulates seladin-1 expression via ERα. Upregulation of seladin-1 probably occurs via classical mechanisms because the seladin-1 gene has an estrogen response element (ERE).

Bcl2 (B-cell lymphoma 2) protein family members can be either pro-apoptotic or anti-apoptotic and together regulate apoptosis via several mechanisms including release of Smac and activation of caspase-9 (Youle and Strasser, 2008). Certain pro-apoptotic Bcl2 family members, such as Bim, can block the anti-apoptotic activity of other Bcl2 family members (Wilson-Annan et al., 2003). Estrogen increases expression of the anti-apoptotic protein BclxL (Bclextra-long) *in vitro*, and this inhibits caspase-mediated proteolysis due to Aβ (Pike, 1999). The estrogen-induced increase in BclxL probably occurs via classical estrogen gene expression regulation because the BclxL gene has a putative ERE, and because the estrogen-induced inhibition of caspase-mediated proteolysis is prevented by ER blockers.

*In vivo*, Aβ activates JNK and also downregulates the anti-apoptotic Bclw and BclxL but upregulates the pro-apoptotic Bim on both the mRNA and protein levels (Yin et al., 2002; Yao et al., 2005, 2007). Bclw and BclxL downregulation, and Bim upregulation, probably results from activation of cJun by JNK. Also, Bclw and BclxL downregulation, and Bim upregulation, are associated with release of Smac, which results in activation of caspase-9. RNA knockdown of Bclw did not lead to release of Smac in the absence of Aβ, while Bim knockdown substantially reduced but did not completely eliminate Smac release, suggesting that Aβ causes Smac release through some combination of different effects. Also *in vivo*, estrogen upregulates Bclw and downregulates Bim at both the mRNA and protein levels (Yao et al., 2007). Estrogen reduces Aβ-induced phosphorylation of JKN, which reduces but does not eliminate the effects of activated JNK on Bclw downregulation and Bim upregulation. It is still uncertain whether estrogen regulates Bclw and Bim via classical ER mechanisms or via signaling pathways. BclxL is probably regulated via classical ER mechanisms (see previous paragraph).

Estrogen increases superoxide dismutase (SOD1 or simply SOD) expression *in vitro* (Rao et al., 2011). The SOD so produced is effective in limiting damage due to OS. The estrogen-induced increase in SOD is believed to occur via ERα.

The mRNA for apoE fluctuates with cyclic changes in estrogen levels in parts of rat hippocampus and hypothalamus (Stone et al., 1997). Estrogen administration increases apoE mRNA and apoE protein in mouse and rat brain regions including cortex and hippocampus, but amounts of apoE mRNA upregulated by estrogen seem to depend on animal strain (Srivastava et al., 1996; Levin-Allerhand et al., 2001; Wang et al., 2006). Selective activation of ERα upregulated apoE while selective activation of ERβ downregulated apoE *in vitro* (Wang et al., 2006). It seems that ERα and ERβ exert antagonistic effects but that ERα overpowers ERβ since estrogen itself upregulates apoE. Administration of estrogen in ovariectomized mice significantly increased LRP in brain regions including hippocampus and cortex (Cheng et al., 2007). The increase in LRP seems to be independent of increases in apoE due to estrogen but the mechanism of LRP increase is unknown. Estrogen also upregulates Pgp expression (Abuznait et al., 2011).

Activation by estrogen either of ERα or ERβ upregulates expression of NEP mRNA and NEP protein *in vitro* (Liang et al., 2010). Activated ERα or ERβexert their influence mainly at EREs on the NEP gene. Activation of ERα or ERβ by selective drugs increases NEP expression less than estrogen itself, indicating that the effects of ERα and ERβ are roughly additive.

### Computational representation and analysis

**Figure 1:**
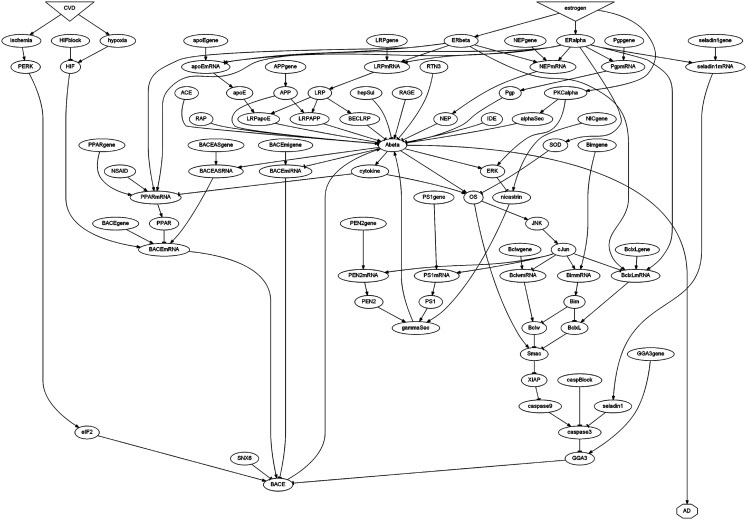
**Schematic diagram of the estrogen-Aβ model**. Model elements are represented using labels within geometric shapes (nodes). Each connection (link) leads from an origin element to a destination element whose level is influenced by that origin element. Arrowhead or tee endings represent positive or negative influence, respectively. Estrogen and cerebrovascular disease (CVD) are the main source elements, but any element with no connections leading to it is a source element. Alzheimer Disease (AD), with no connections leading from it, is the only sink element. The other element labels are defined in the text.

The interactions outlined in the previous two subsections are diagrammed in Figure 1, and are represented computationally in a computer program. The diagram is a directed graph, in which the graph elements are represented as nodes while their interactions are represented as links. Each node has a label representing a molecular species (e.g., Aβ) or condition (e.g., OS). Each node has a variable, numerical value associated with it, and that value is meant to correspond to an experimentally measureable quantity. However, due to the qualitative nature of the data on which the model is based, the correspondence between numerical variable values and experimental measurements is intended to be proportional rather than exact. To alleviate any ambiguity, node labels correspond to the actual variable names used in the computer program, emphasizing the fact that all reported numerical results directly pertain to computer model variables rather than experimental measurements. Node names reflect accepted nomenclature as closely as possible within the symbolic limitations of the programming language. Among other limitations, the programming language does not support Greek lettering, dashes, or spaces in element names, so accepted nomenclature was altered accordingly (e.g., Abeta, BACEmRNA, caspase3). To further distinguish model elements from the biological entities they represent, all element names are printed in monotype font (e.g., Abeta, BACEmRNA, caspase3).

Most nodes in the diagram are represented as ovals. Internal nodes both receive and send links, and all internal elements are represented as oval nodes. Source elements are those with no incoming links. The two main source elements, represented as inverted triangles, are estrogen and CVD, but many other source elements are represented as ovals. The single sink node, which sends no links, is AD, represented as an octagon. Links represent influence rather than binding. Thus, ERα activation increases seladin-1 mRNA expression by binding an ERE on the seladin-1 gene (see previous two subsections of Materials and Methods for references), but this binding is not represented in the model. Instead, the influence is represented by having ERalpha and seladin1gene each send a link to seladin1mRNA, since both ERalpha and seladin1gene influence seladin1mRNA expression.

Links are either positive (arrowhead ending) or negative (tee ending). Due to the qualitative nature of the data, all links have absolute value one unless information is available to specify a different value. This occurs in two places in the model. First, one molecule of LRP binds one molecule of APP so LRP APP binding helps produce one molecule of Aβ, but one molecule of apoE can bind multiple molecules of Aβ and so clear multiple Aβ molecules after the LRP-apoE complex is internalized. Thus, the link from LRPAPP to Abeta has value +1 but, parsimoniously, the link from LRPapoE to Abeta has value −2. Second, activation of ERα and ERβ respectively increase and decrease apoE mRNA expression, but estrogen, which activates both ERs, produces a net increase in apoE mRNA expression. Thus, the link from ERalpha to apoEmRNA has value +2 while the link from ERbeta to apoEmRNA has value −1.

The interactions depicted in the diagram are all represented in a computer program written in a specialized meta-language known as Maude (Clavel et al., 2007). Maude is a declarative programming language in which statements are declarations of facts rather than imperative commands, and for that reason Maude programs are also referred to as specifications. The main difference between the two computational approaches is that in an imperative program, commands execute in a specified order while in a specification, declarations execute in an unspecified order provided they are applicable. Both imperative and declarative programming languages can be used to create computer models of real systems but, because the statements in a specification are declarations of facts, a declarative specification can be used not only to simulate a system but also to analyze it using the methods of temporal-logic (Huth and Ryan, 2004). Both simulation and temporal-logic analysis will be applied here in evaluating the model of the contribution of estrogen to Aβ regulation (called the estrogen-Aβ model).

A specification in Maude is based on an underlying algebra characterized by a set of data types and allowed operations on, and between, those data types (Clavel et al., 2007). Each element in the estrogen-Aβ model is represented by an operator that assigns an integer value to that element. For example, the operator estrogen(1) assigns the integer value 1 to estrogen and makes that assignment a State of the system. A concatenation operator allows multiple State expressions to be combined into a single State expression. Some other operators serve only as integer constants that are used as thresholds or levels of constitutive expression. Specifically, the operators thrCY, thrERK, thrOS, and thrAD are the input thresholds for activation of cytokine, ERK, OS, and AD and they take integer values of 6, 6, 9, and 12, respectively. The operators conBACEmRNA, conBACE, and conAbeta assign integer baselines (i.e., constitutive expression levels) of 6 to each of BACEmRNA, BACE, and Abeta. These thresholds and levels of constitutive expression, combined with the interaction (i.e., link) values described above, constitute the full set of parameters of the model. Using these parameters, the interactions diagrammed in Figure 1, and the interaction descriptions provided in the previous two subsections of Materials and Methods, the modeling results presented in Results should be reproducible.

Declarations in Maude are either equations or rules, and together they describe the system being modeled. Both equations and rules are based on the underlying algebra and its data types and allowed operations. The main difference between them is that applicable equations must execute, but applicable rules may execute or not. This difference is critical for the ability of Maude to analyze a system because she can follow not just one but every possible order of applicable rule executions and thereby explore the entire model state space. On each path through the state space, Maude executes an applicable rule and then executes every equation made applicable by execution of that rule. She then executes another applicable rule, but the order of rule executions is different for each path. By constructing and then searching all possible paths through the state space, Maude can evaluate temporal-logic propositions such as whether one specific state always leads to another specific state, or whether one specific state pertains only until another specific state occurs, and so on (Huth and Ryan, 2004; Clavel et al., 2007).

The integer values assigned by the operators to model elements are kept non-negative. For many elements, this is accomplished by treating their integer levels simply as binary (i.e., their levels are of the integer data type but they are restricted to values of 0 or 1). Other elements are allowed to take multiple integer levels but they are kept non-negative through logic and/or by providing one of the baseline, constitutive levels of expression so that negative inputs do not force their levels to go below zero. Source nodes, which receive no inputs, have their levels set at the beginning of a simulation or analysis. Through the many forward and recurrent (i.e., feedback) links in the model, whose effects are described by rules and equations, the levels of the source and internal nodes affect the levels of the other internal nodes and, ultimately, the sink node.

The Maude specification for the estrogen-Aβ model is called ALZHEIMERE, where the last E stands for estrogen. Each declaration in ALZHEIMERE describes how the level of one element is affected by the levels of its input elements. Only three declarations in the model are rules. These are the declarations that describe the activation of ERalpha, ERbeta, and PKCalpha by estrogen. For example, Maude code for the declaration that determines the activation of ERalpha is shown below (all Maude code is written in monotype font).



crl [ERalphaAct]:estrogen(X1) ERalphaAg(X2)
         ERalpha(Y) =>
         estrogen(X1) ERalphaAg(X2)
         ERalpha(if X1 > 0 or
          X2 > 0 then 1 else 0 fi)
         if Y =/= if X1 > 0 or
          X2 > 0 then 1 else 0 fi.



In this rule, called ERalphaAct, the levels of estrogen, the ERα agonist ERalphaAg, and ERalpha are represented by integer variables X1, X2, and Y, respectively. The rule specifies that the levels of estrogen and ERalphaAg remain the same but the level of ERalpha goes to 1 if either X1 or X2 is greater than 0, or goes to 0 if neither X1 nor X2 is greater than 0. Note that the rule is conditional (crl), so that the level of ERalpha can *change* to 1 or 0 but will not be reassigned 1 or 0 if it had that value already. The conditionality of the rule makes it applicable only if executing it will change the value assigned to ERalpha. Note also that the conditional rule limits the value of ERalpha to binary values. The conditional rules that determine the activation of ERbeta and PKCalpha (not shown) are similar.

All of the other declarations in ALZHEIMERE are equations and, more specifically, conditional equations. For example, the conditional equation (ceq) that determines the level of BACEmRNA expression, called BACEmessage, is shown below.



ceq [BACEmessage]:BACEgene(G) BACEASRNA(X1)
         HIF(X2) PPAR(Y)
         BACEmRNA(Z) =
         BACEgene(G) BACEASRNA(X1)
         HIF(X2) PPAR(Y)
         BACEmRNA(((conBACEmRNA
          + X1 + X2) - Y) * G)
         if Z =/= ((conBACEmRNA
          + X1 + X2) - Y) * G.



In this equation the levels of BACEgene, BACEASRNA, HIF, PPAR, and BACEmRNA are represented by the integer variables G, X1, X2, Y, and Z, respectively. The equation specifies that the levels of BACEgene, BACEASRNA, HIF, and PPAR remain unchanged but the level of BACEmRNA equals the sum of its positive inputs X1 and X2 and its constitutive level conBACEmRNA, minus its negative input Y, and this difference is multiplied by G, which is 1 if the gene is present but 0 if it is absent. The conditionality of the equation makes it applicable only if executing it will change the value assigned to BACEmRNA.

The rules and equations need to be conditional to prevent them from executing if doing so would not change the value of any element. Without such a condition, the declaration that determines the level of any element, whether equation or rule, would continue executing ad infinitum, simply replacing the value assigned by its operator with the identical value. With such conditions, declarations do not execute unless doing so would change the value of an element, and the specification terminates when no further changes to element values can be made. The Maude specification ALZHEIMERE terminates for all start configurations (i.e., assignments of source node values) that are reported in Results.

In the context of declarative programming, temporal-logic analysis is also known as model checking. Model checking for Maude specification ALZHEIMERE is carried out using a separate specification called MC-ALZHEIMERE. The main function of MC-ALZHEIMERE is to define a set of properties of interest that can be verified through executions of rules and equations in ALZHEIMERE. For example, the following (non-conditional) equation indicates when the property NEPeq3 is true.



eq AM(S NEP(3)) |= NEPeq3 = true.



(where | = is the logical connector “satisfies”). The operator AM is a “wrapper” and the term AM(S NEP(3)) simply defines the state of the system when NEP is at level 3 and all other elements, at whatever levels, are subsumed under the variable S. The equation specifies that the system with NEP at level 3 satisfies the proposition that NEPeq3 is true, regardless of other element levels. In executing this equation in MC-ALZHEIMERE, Maude will determine, given some initial configuration of the model (i.e., assignments of source node values) and rule and equation executions in ALZHEIMERE, that NEPeq3 is true, or it will be unable to execute further rules and equations in ALZHEIMERE and will “deadlock” without determining that NEPeq3 is true. Temporal-logic analysis involves verification of properties individually or combined into more complex logical statements. Both simulation and temporal-logic analysis will be used to generate results on, and to derive predictions from, the estrogen-Aβ model.

## Results

The Maude specification ALZHEIMERE computationally describes the interactions depicted in Figure 1, which represent many of the recently identified contributions that estrogen makes to the regulation of Aβ. By representing these interactions the Maude model can indicate what they mean in the aggregate. More specifically, the model can indicate what effects different combinations of hormone or estrogen receptor agonists and other drugs might have on the level of Aβ, whether under conditions of low estrogen alone or exacerbated by mild CVD. The Maude model, like any model, is a hypothesis that needs to be verified experimentally, and many of the results of model simulation and analysis reported in this section can be taken as predictions of the model (see Discussion).

Simulations begin by finding the normal, baseline level of Aβ in the model. This is done by setting all of the source elements to their normative levels (including the normative level of estrogen) and then letting Maude execute all applicable equations and rules until a terminal state is reached. In Maude, a rule execution is known as a “rewrite” while an equation execution is known as a “reduction.” Since rule executions (i.e., rewrites) can make equations applicable and so lead to equation executions (i.e., reductions), both rule and equation executions can be produced using rewrite commands. A command to produce twenty rewrites in the Maude specification ALZHEIMERE from the normative start-state is shown below.



rewrite [20] in ALZHEIMERE : PPARgene(1)
PS1gene(1) PEN2gene(1) NICgene(1)
seladin1gene(1) GGA3gene(1) APPgene(1)
LRPgene(1) apoEgene(1) Pgpgene(1)
BACEASgene(1) BACEmigene(1) BACEgene(1)
NEPgene(1) BclxLgene(1) Bclwgene(1)
Bimgene(1) estrogen(1) SNX6(1) RAGE(1)
hepSul(1) RTN3(1) RAP(1) IDE(1) ACE(1)
CVD(0) NSAID(0) HIFblock(0) caspBlock(0)
ERKblock(0) PERKblock(0) ERalphaAg(0)
ERbetaAg(0) hypoxia(0) HIF(0) ischemia(0)
PERK(0) eIF2(0) cytokine(0) PPARmRNA(0)
PPAR(0) OS(0) JNK(0) cJun(0) SOD(0) ERK(0)
PS1mRNA(0) PS1(0) PEN2mRNA(0) PEN2(0)
nicastrin(0) gammaSec(0) PKCalpha(0)
alphaSec(0) Smac(0) XIAP(0) caspase9(0)
caspase3(0) seladin1mRNA(0) seladin1(0)
GGA3(0) APP(0) Abeta(0) AD(0) LRPmRNA(0)
LRP(0) apoEmRNA(0) apoE(0) LRPapoE(0)
LRPAPP(0) BECLRP(0) PgpmRNA(0) Pgp(0)
BACEASRNA(0) BACEmiRNA(0) BACEmRNA(0)
BACE(0) NEPmRNA(0) NEP(0) BclxLmRNA(0)
BclxL(0) BclwmRNA(0) Bclw(0) BimmRNA(0)
Bim(0) ERalpha(0) ERbeta(0)



The order of operators in this list is arbitrary and is, in any case, determined internally by Maude. In the normative start-state, all genes (e.g., APPgene) and all endogenous factors (e.g., SNX6) are present, but all agonists (e.g., ERalphaAg) and other drugs (e.g., NSAID) are absent. Also in the normative state estrogen is present but CVD is absent. Note that a source element is present or absent when its level is 1 or 0, respectively. In the start-state the levels of all of the internal nodes, including Abeta, and the sink node AD, are set to 0. Twenty rewrites is more than enough to ensure that all applicable rules will execute. The result of rewriting from the normative start-state in Maude specification ALZHEIMERE is shown below.



result State: CVD(0) hypoxia(0) HIF(0)
HIFblock(0) ischemia(0) PERK(0) PERKblock(0)
eIF2(0) cytokine(0) PPARgene(1) PPARmRNA(2)
PPAR(2) NSAID(0) OS(0) JNK(0) cJun(0) SOD(1)
ERK(0) ERKblock(0) PS1gene(1) PS1mRNA(1)
PS1(1) PEN2gene(1) PEN2mRNA(1) PEN2(1)
NICgene(1) nicastrin(2) gammaSec(1)
PKCalpha(1) alphaSec(1) Smac(0) XIAP(0)
caspase9(0) caspase3(0) caspBlock(0)
seladin1gene(1) seladin1mRNA(1) seladin1(1)
GGA3gene(1) GGA3(2) SNX6(1) APPgene(1)
APP(1) Abeta(2) AD(0) LRPgene(1) LRPmRNA(2)
LRP(2) apoEgene(1) apoEmRNA(2) apoE(2)
LRPapoE(2) LRPAPP(1) BECLRP(2) Pgpgene(1)
PgpmRNA(2) Pgp(2) RAGE(1) BACEASgene(1)
BACEASRNA(2) BACEmigene(1) BACEmiRNA(1)
BACEgene(1) BACEmRNA(6) BACE(8) hepSul(1)
RTN3(1) RAP(1) IDE(1) ACE(1) NEPgene(1)
NEPmRNA(3) NEP(3) BclxLgene(1) BclxLmRNA(2)
BclxL(2) Bclwgene(1) BclwmRNA(1) Bclw(1)
Bimgene(1) BimmRNA(1) Bim(1) estrogen(1)
ERalpha(1) ERbeta(1) ERalphaAg(0)
ERbetaAg(0)



Again the order of operators in the list is arbitrary. The levels of the source elements remain unchanged while the levels of many, but not all, of the internal elements have changed. Note, for example, that with CVD absent, neither hypoxia nor ischemia occurs. Consequently, neither HIF nor PERK is activated. With estrogen present, both ERalpha and ERbeta are activated. Also, with CVD absent and estrogen present, OS does not occur and cytokine, JNK, ERK, and caspase3 are not activated. In this, the normative baseline state, the heavily regulated BACEmRNA, BACE, and Abeta take integer values of 6, 8, and 2, respectively, and AD does not occur. These normative results are shown in Row 1 of Table 1. It is imperative to reiterate (see Computational Representation and Analysis) that the levels of the various model elements are meant to be proportional to levels of real molecules, and that changes in model element levels due to changes in model inputs constitute the relevant modeling results.

**Table 1:** **Simulating the effects of estrogen and its lack, and of selective ERα and ERβ agonists, on BACEmRNA, BACE, and Aβ levels in the absence of CVD in the model**.

Number	Estrogen	ERalphaAg	ERbetaAg	CVD	NSAID	HIFblock	caspBlock	ERalpha	ERbeta	OS	caspase3	BACEmRNA	BACE	Abeta	AD
1	1	0	0	0	0	0	0	1	1	0	0	6	8	2	0
2	0	0	0	0	0	0	0	0	0	1	1	8	11	11	0
3	0	0	0	0	1	0	0	0	0	1	1	7	10	10	0
4	0	0	0	0	0	1	0	0	0	1	1	8	11	11	0
5	0	0	0	0	0	0	1	0	0	1	0	8	10	10	0
6	0	0	0	0	1	0	1	0	0	1	0	7	9	9	0
7	0	0	0	0	1	1	1	0	0	1	0	7	9	9	0
8	0	0	1	0	0	0	0	0	1	1	1	7	10	11	0
9	0	0	1	0	1	0	0	0	1	1	1	6	9	10	0
10	0	0	1	0	0	1	0	0	1	1	1	7	10	11	0
11	0	0	1	0	0	0	1	0	1	1	0	7	9	10	0
12	0	0	1	0	1	0	1	0	1	1	0	6	8	9	0
13	0	0	1	0	1	1	1	0	1	1	0	6	8	9	0
14	0	1	0	0	0	0	0	1	0	0	0	6	8	4	0
15	0	1	0	0	1	0	0	1	0	0	0	5	7	3	0
16	0	1	0	0	0	1	0	1	0	0	0	6	8	4	0
17	0	1	0	0	0	0	1	1	0	0	0	6	8	4	0
18	0	1	0	0	1	1	1	1	0	0	0	5	7	3	0

*Each row shows the consequences of a specific start configuration (the seven source elements heading the columns on the left) for a selected set of elements of interest (the eight elements heading the columns on the right). The row numbers appear in the leftmost column*.

### Lowering Aβ in the absence of estrogen and the absence of CVD

Table 1 tabulates the results of model simulations (i.e., rewrites in ALZHEIMERE) initiated from many different start configurations but all in the absence of CVD. Whereas Row 1 of Table 1 shows the normal, baseline state of the model, Row 2 shows the consequences for the model of removing estrogen. Neither ERalpha nor ERbeta are activated in the absence of estrogen and in the absence of any estrogen receptor agonists. This lack leads to the activation of OS and caspase3. That and other changes in internal element levels lead to increases of BACEmRNA from 6 to 8, of BACE from 8 to 11, and of Abeta from a normative level of 2 to 11. Because the model is focused on Aβ regulation, and because the actual relationship between Aβ and AD is still incompletely understood, the Aβ threshold at which AD occurs is set arbitrarily in the model. This threshold is set at the relatively high level of 12, and AD occurs only if Abeta is strictly greater than 12, so AD does not occur in the absence of both estrogen and CVD (Row 2 of Table 1). The more important finding in this case is the change in Abeta, and the increase in Abeta from 2 to 11 is substantial in the context of the model.

Considering the many ways in which estrogen limits Abeta production in the model, this drastic rise in Abeta level in the absence of estrogen is not surprising, even without CVD. Through the ERalpha and ERbeta receptors, estrogen reduces BACEmRNA expression by increasing PPAR expression (via upregulation of PPARmRNA), and the decrease in BACEmRNA leads to a decrease in BACE and so in Abeta. Also through its classical receptors, estrogen decreases Abeta by increasing expression of LRP, apoE, NEP, and Pgp. Less directly, estrogen reduces Abeta because it reduces OS by upregulating SOD, and suppresses caspase3 activation by upregulating seladin1 and BclxL. Because OS and caspase3 activation can augment BACE levels, estrogen reduces BACE and so reduces Abeta by reducing OS and caspase3 activation. Through its activation of PKCalpha, estrogen further reduces Abeta by activating alphaSec, which diverts APP away from the amyloidogenic pathway. Due to the presence of numerous positive feedback loops by which Abeta upregulates BACE and so drives its own accumulation, estrogen can, essentially, reduce Abeta by reducing this positive feedback drive. Thus, estrogen exerts downward pressure on Abeta in about 10 different ways in the model, and so the dramatic upraising of Abeta in the absence of estrogen is not surprising.

The rest of Table 1 is concerned with the question of how various drugs, alone or in combination, could help keep Abeta levels down in the absence of estrogen (the state of 0 estrogen in the model corresponds to a worst-case post-menopausal scenario). In the model the effect of NSAID is to augment the expression of PPARmRNA and so of PPAR itself (other possible effects of NSAIDs are not represented for simplicity). Because PPAR suppresses BACEmRNA expression, NSAID reduces BACEmRNA and, consequently, also reduces BACE and Abeta. With links of absolute value 1 for the interactions that mediate these NSAID effects, activation of NSAID (i.e., changing NSAID level from 0 to 1) results in a decrease of 1 for each of BACEmRNA, BACE, and Abeta (compare Rows 3 and 2 of Table 1).

The function of HIFblock in the model is to keep HIF at level 0 even in the presence of hypoxia. In the model, hypoxia is brought about by CVD and hypoxia does not occur without it. For the simulations whose results are listed in Table 1, CVD is absent, hypoxia does not occur, and HIF is not activated, so blocking HIF using HIFblock should have no effect. Indeed it does not (compare Rows 4 and 2 of Table 1). The presence of HIFblock does not reduce Abeta from its high level of 11 in the absence of estrogen.

The function of caspBlock in the model is, likewise, to keep caspase3 at level 0 despite activation of the pro-apoptotic pathway. This pathway is activated by OS in the model and is regulated by many factors, some of which are under the control of estrogen (e.g., BclxL and seladin1). The occurrence of OS in the model is determined by the level of Abeta and by the activation of cytokine, which itself depends on the level of Abeta, and the rise in Abeta resulting from the absence of estrogen is enough to cause OS and so activate caspase3. In this case blocking caspase3 using caspBlock should have a beneficial effect. Indeed it does (compare Rows 5 and 2 of Table 1). Note that caspase3 exerts its effects on BACE rather than on BACEmRNA, so the presence of caspBlock does not change the level of BACEmRNA but it decreases the levels both of BACE and of Abeta by 1.

Two factors that independently produce the same effect could produce a greater effect in combination, especially if they work via different pathways. This is the case for NSAID and caspBlock (compare Rows 6 and 2 of Table 1). Applying NSAID and caspBlock together reduces BACEmRNA from 8 to 7, BACE from 11 to 9, and Abeta from 11 to 9. Since HIF is activated only by CVD and CVD is not present in this set of simulations, HIFblock, even in combination with other factors, should provide no benefit. As expected, in the absence of both estrogen and CVD, HIFblock in combination with NSAID and caspBlock is not more effective in reducing Abeta than are NSAID and caspBlock without HIFblock (compare Rows 7 and 6 of Table 1). These simulations suggest than in a state of very low estrogen, the rise in Aβ could be slightly offset using an NSAID-class drug that promotes PPAR expression or a compound that blocks caspase-3 activation, and that their combination would be more effective than either one alone. This set of simulations further suggests that, in the absence of CVD, compounds that block HIF activation would not be effective in lowering Aβ levels consequent to estrogen depletion, even in combination with other compounds that work via different pathways.

An alternative to estrogen therapy is to administer drugs that act as agonists for specific estrogen receptors. This alternative is explored computationally using the model, and the results are tabulated in the second and third blocks of Table 1. Again, both estrogen and CVD are absent. The simulations show that the administration of an agonist of ERβ (ERbetaAg) provides no benefit in the model. The effect of ERbetaAg alone is to decrease BACEmRNA from 8 to 7 and to decrease BACE from 11 to 10, but ERbetaAg alone does not decrease Abeta (compare Rows 8 and 2 of Table 1). This initial result exposes the main drawback of ERbetaAg. Although it provides some of the same benefits as estrogen, ERbetaAg causes a decrease in apoE. Specifically, apoE is expressed at level 1 without estrogen but at level 0 with the addition of ERbetaAg. Because ERbetaAg suppresses apoE it cannot lower Abeta despite lowering BACEmRNA and BACE. In the model, ERbetaAg provides no benefit by itself.

As was the case previously without estrogen and without CVD, NSAID and caspBlock both reduce Abeta in the presence of ERbetaAg but HIFblock does not. Again as in the previous case, NSAID and caspBlock work better together than separately but HIFblock remains ineffective even in combination with NSAID and caspBlock (see Rows 9 through 13 in Table 1). Because it reduces the level of apoE, ERbetaAg provides no benefit over NSAID and caspBlock despite lowering both BACEmRNA and BACE. The model suggests that ERβ agonists would not be effective replacements for estrogen for the purpose of Aβ reduction.

The situation is dramatically different with administration of an ERα agonist (ERalphaAg). The presence of ERalphaAg alone reduces BACEmRNA from 8 to 6, BACE from 11 to 8, and Abeta from 11 to 4 (compare Rows 14 and 2 of Table 1). There are three reasons why ERalphaAg alone reduces Abeta substantially while ERbetaAg alone does not reduce Abeta at all. First, ERalphaAg increases apoE while ERbetaAg decreases it. Specifically, apoE is expressed at level 1 without estrogen, is expressed at level 0 with the addition of ERbetaAg alone, but is expressed at level 3 with the addition of ERalphaAg alone. Second, ERalphaAg increases seladin1, but ERbetaAg has no effect on it. Third, the combined effects of ERalphaAg, including increases in apoE, seladin1, NEP, and Pgp, are enough to bring Abeta below the threshold at which it activates cytokine and OS. By preventing OS in the absence of estrogen, ERalphaAg breaks the main, pathological positive feedback loop by which Abeta drives its own accumulation, and that keeps Abeta levels down.

As before in the absence of estrogen, or with ERbetaAg, NSAID is also able to reduce each of BACEmRNA, BACE, and Abeta by 1 in combination with ERalphaAg (compare Rows 15 and 14 of Table 1). Again as before in the absence of CVD, HIFblock is ineffective in reducing Abeta (or BACEmRNA or BACE) with ERalphaAg because HIF is not activated unless CVD is present to cause hypoxia (compare Rows 16 and 14 of Table 1). Because ERalphaAg, like estrogen itself, increases seladin1, and because seladin1 suppresses caspase3, caspBlock is also ineffective in reducing Abeta (or BACEmRNA or BACE) with ERalphaAg (compare Rows 17 and 14 of Table 1). Thus, in the absence of estrogen and CVD, a combination of ERalphaAg and NSAID is capable of bringing Abeta back down to 3, which is close to the normative level of 2 (compare Rows 18 and 1 of Table 1). The model suggests that, in the post-menopausal state of low estrogen, the level of Aβ could be reduced almost as effectively with a combination of an ERα agonist and an NSAID as with estrogen itself.

### Lowering Aβ in the presence of CVD

Lack of estrogen leads to a pronounced upraising of Abeta in the model, as described in the previous subsection and as shown in Rows 1 and 2 of Table 1. This is exacerbated by CVD, as shown in Row 1 of Table 2. In the absence of estrogen but in the presence of CVD, BACEmRNA rises from a normative level of 6 to 9, BACE from a normative 8 to 13, and Abeta from a normative 2 to 13 (compare Row 1 of Table 1 with Row 1 of Table 2). With the AD threshold set arbitrarily at 12 in the model, the absence of estrogen combined with the presence of CVD raises Abeta enough to cause AD. The obvious implication here is that CVD would be expected to exacerbate the Aβ accumulation due to low estrogen, especially since CVD raises Aβ via pathways different from those by which estrogen lowers it (see Figure 1).

**Table 2:** **Simulating the effects of estrogen and its lack, and of selective ERα and ERβ agonists, on BACE mRNA, BACE, and Aβ levels in the presence of CVD in the model**.

Number	Estrogen	ERalphaAg	ERbetaAg	CVD	NSAID	HIFblock	caspBlock	ERalpha	ERbeta	OS	caspase3	BACEmRNA	BACE	Abeta	AD
1	0	0	0	1	0	0	0	0	0	1	1	9	13	13	1
2	0	0	0	1	1	0	0	0	0	1	1	8	12	12	0
3	0	0	0	1	0	1	0	0	0	1	1	8	12	12	0
4	0	0	0	1	0	0	1	0	0	1	0	9	12	12	0
5	0	0	0	1	1	1	0	0	0	1	1	7	11	11	0
6	0	0	0	1	1	0	1	0	0	1	0	8	11	11	0
7	0	0	0	1	0	1	1	0	0	1	0	8	11	11	0
8	0	0	0	1	1	1	1	0	0	1	0	7	10	10	0
9	0	0	1	1	0	0	0	0	1	1	1	8	12	13	1
10	0	0	1	1	1	0	0	0	1	1	1	7	11	12	0
11	0	0	1	1	0	1	0	0	1	1	1	7	11	12	0
12	0	0	1	1	0	0	1	0	1	1	0	8	11	12	0
13	0	0	1	1	1	1	0	0	1	1	1	6	10	11	0
14	0	0	1	1	1	0	1	0	1	1	0	7	10	11	0
15	0	0	1	1	0	1	1	0	1	1	0	7	10	11	0
16	0	0	1	1	1	1	1	0	1	1	0	6	9	10	0
17	0	1	0	1	0	0	0	1	0	0	0	8	11	7	0
18	0	1	0	1	1	0	0	1	0	0	0	6	9	5	0
19	0	1	0	1	0	1	0	1	0	0	0	6	9	5	0
20	0	1	0	1	0	0	1	1	0	0	0	8	11	7	0
21	0	1	0	1	1	1	0	1	0	0	0	5	8	4	0
22	0	1	0	1	1	1	1	1	0	0	0	5	8	4	0
23	1	0	0	1	0	0	0	1	1	0	0	7	10	4	0
24	1	0	0	1	1	0	0	1	1	0	0	6	9	3	0
25	1	0	0	1	0	1	0	1	1	0	0	6	9	3	0
26	1	0	0	1	0	0	1	1	1	0	0	7	10	4	0
27	1	0	0	1	1	1	0	1	1	0	0	5	8	2	0
28	1	0	0	1	1	1	1	1	1	0	0	5	8	2	0

*Each row shows the consequences of a specific start configuration (the seven source elements heading the columns on the left) for a selected set of elements of interest (the eight elements heading the columns on the right). The row numbers appear in the leftmost column*.

As before, in the absence of both estrogen and CVD (Rows 3 and 5 of Table 1), NSAID and caspBlock are also effective in lowering Abeta in the absence of estrogen but in the presence of CVD (Rows 2 and 4 of Table 2). Furthermore, because CVD, and the hypoxia it causes are present, HIFblock is effective in lowering Abeta in this case (Row 3 of Table 2) whereas it was not in the case of no CVD (Row 4 of Table 1). Also with estrogen absent but CVD present, NSAID, HIFblock, and caspBlock are more effective in combination than separately (Rows 5 through 8 of Table 2). Specifically, in the absence of estrogen but in the presence of CVD, each of NSAID, HIFblock, and caspBlock can lower BACE and Abeta by 1 and their effects are additive. Although each of NSAID and HIFblock can lower BACEmRNA by 1, caspBlock has no effect on BACEmRNA. The lack of effect of caspBlock on BACEmRNA results because caspase3 bypasses BACEmRNA and works directly to increase BACE by suppressing GGA3. These simulations suggest than in a state of very low estrogen combined with incipient CVD, the rise in Aβ could be slightly offset using an NSAID-class drug that promotes PPAR expression, or a compound that blocks HIF, or a compound that blocks caspase-3 activation, and that administration of any two, or all three, of the compounds in combination would be more effective than any one of the three administered alone.

Although the level of Abeta is a bit higher with estrogen absent but CVD present, the effects of ERbetaAg in this case (Rows 9 through 16 of Table 2) are similar to those with estrogen and CVD both absent (Rows 8 through 13 of Table 1) in the sense that ERbetaAg can lower BACEmRNA by 1, and thereby lower BACE by 1, but it has no net effect on Abeta. As with estrogen absent but CVD present (Rows 1 through 8 of Table 2), each of NSAID, HIFblock, and caspBlock can lower BACE and Abeta by 1 and their effects are additive, and NSAID and HIFblock lower BACE by lowering BACEmRNA while caspBlock lowers BACE without also lowering BACEmRNA (Rows 9 through 16 of Table 2). Because ERbetaAg, like estrogen itself, reduces BACEmRNA via PPAR, both BACEmRNA and BACE are lower, under all combinations of NSAID, HIFblock, and caspBlock, in the presence of ERbetaAg when estrogen is absent but CVD is present (compare Rows 9 through 16 with Rows 1 through 8 of Table 2). However, because ERbetaAg administered in the absence of estrogen reduces apoE from 1 to 0, the net effect of ERbetaAg on Abeta is 0. The implication of these modeling results is that, under conditions of low estrogen and incipient CVD, administration of an ERβ agonist would provide no benefit in terms of lowering Aβ.

In sharp contrast to the overall lack of benefit of ERbetaAg, administration of ERalphaAg provides a substantial benefit in terms of lowering Abeta with estrogen absent but CVD present (Rows 17 through 22 of Table 2), as it had previously with estrogen and CVD both absent (Rows 14 through 18 of Table 1). Addition of ERalphaAg alone reduces BACEmRNA from 9 to 8, BACE from 13 to 11, and Abeta from 13 to 7 (compare Rows 17 and 1 of Table 2). The sharp drop in Abeta occurs because the suppression of Abeta production, and the promotion of Abeta elimination, due to ERalphaAg are enough to prevent Abeta from rising about the threshold for activation of OS, and this breaks the main, pathological positive feedback loop by which Abeta drives its own accumulation in the model. OS is among the factors that activate caspase3, while seladin1 is among the factors that inactivate it, so by preventing OS and promoting seladin1, ERalphaAg is able to suppress caspase3. For this reason, caspBlock does not provide additional benefit in terms of lowering Abeta when ERalphaAg is added under conditions in which estrogen is absent but CVD is present (compare Rows 20 and 17 of Table 2).

Although caspBlock is ineffective in combination with ERalphaAg under conditions in which estrogen is absent but CVD is present, each of NSAID or HIFblock is even more effective in combination with ERalphaAg than either is alone (compare Rows 17 through 19 with Rows 1 through 3 in Table 2). Specifically, with estrogen absent but CVD present, NSAID or HIFblock alone reduces Abeta from 13 to 12 (Rows 1 through 3 of Table 2), but in combination with ERalphaAg, NSAID, or HIFblock reduces Abeta from 7 to 5 (Rows 17 through 19 of Table 2). Thus, with estrogen absent but CVD present, NSAID or HIFblock alone reduces Abeta by 1, but either reduces Abeta by 2 in combination with ERalphaAg (compare Rows 17 through 19 with Rows 1 through 3 in Table 2). The reason for the larger drop is that NSAID or HIFblock in combination with ERalphaAg lowers Abeta enough to prevent the activation of cytokine, which indirectly increases BACE and so increases Abeta. This synergistic (more than additive) effect of ERalphaAg combined with either NSAID or HIFblock is due to a threshold effect, specifically a failure to cross the cytokine threshold. Similarly, the dramatic effect of ERalphaAg alone, which reduces Abeta from 11 to 4 with estrogen and CVD both absent (compare Rows 2 and 14 of Table 1), and reduces Abeta from 13 to 7 with estrogen absent but CVD present (compare Rows 1 and 17 of Table 2), is also due to a threshold effect, specifically a failure to cross the OS threshold.

Under conditions in which estrogen is absent but CVD is present, and ERalphaAg is administered in combination either with NSAID or HIFblock, neither the OS nor the cytokine thresholds will be crossed, and Abeta will be held at the relatively low level of 5 (Rows 18 and 19 in Table 2). Under these same conditions, administration of ERalphaAg in combination with both NSAID and HIFblock will further reduce Abeta to 4 (Row 21 in Table 2). Administration of caspBlock confers no additional benefit (Row 22 of Table 2). These results suggest that, under conditions of low estrogen and incipient CVD, administration of an ERα agonist alone or combined with an NSAID-class drug that promotes PPAR expression and/or a compound that blocks HIF, would be effective in lowering Aβ, but that a compound that blocks caspase-3 activation would confer no additional benefit.

While ERalphaAg alone, and especially in combination with NSAID and HIFblock, produces a substantial reduction in Abeta when estrogen is absent but CVD is present, administration of estrogen itself is even more effective. Administration of estrogen in the presence of CVD lowers BACEmRNA from 9 to 7, BACE from 13 to 10, and Abeta from 13 to 4 (compare Rows 1 and 23 of Table 2). This dramatic lowering of Abeta is a compound threshold effect, since administration of estrogen in the presence of CVD lowers Abeta below the thresholds of activation of both OS and cytokine. Under these conditions, administration of NSAID or HIFblock results in further reduction of BACEmRNA, BACE, and Abeta each by 1 (Rows 24 and 25 of Table 2). Because estrogen both prevents OS and promotes seladin1 it is able to suppress caspase3, so administration of caspBlock provides no additional benefit in the presence of both estrogen and CVD (Row 26 in Table 2). When Abeta is below both the OS and cytokine thresholds the effects of NSAID and HIFblock are additive, rather than synergistic, so that administration of NSAID and HIFblock together results in a further reduction of BACEmRNA, BACE, and Abeta each by 1 (Row 27 of Table 2). As expected, caspBlock provides no additional benefit in terms of Abeta reduction in the presence of estrogen, even if administered in combination with NSAID and HIFblock (Rows 26 and 28 of Table 2). In the presence of CVD, the combined administration of estrogen, NSAID, and HIFblock brings Abeta from the AD-inducing level of 13 all the way back to the normative level of 2 (compare Rows 1 and 27 of Table 2). These modeling results suggest the possibility that, under conditions of very low estrogen and incipient CVD, the level of Aβ could be brought back to normal with a combination of an NSAID-class drug that promotes PPAR expression and a compound that blocks HIF, in conjunction with estrogen itself.

### ERα agonists may be less effective than estrogen itself in lowering Aβ

Using NSAID and HIFblock in the presence of CVD, ERalphaAg can reduce Abeta to 4, but estrogen itself can reduce Abeta all the way back to the normative level of 2 (compare Rows 21 and 27 of Table 2). Although estrogen by itself can prevent cytokine activation in the presence of CVD, ERalphaAg can also prevent cytokine activation in combination with NSAID and HIFblock (note that estrogen or ERalphaAg alone can prevent OS as shown in Rows 17 and 23 of Table 2). Furthermore, apoE reaches level 2 with estrogen but reaches level 3 with ERalphaAg, so ERalphaAg provides a benefit over estrogen itself in terms of apoE. Why then is estrogen able to reduce Abeta even further than ERalphaAg? Temporal-logic analysis can be used to answer that question in the model.

Temporal-logic analysis begins by defining properties of interest, and then checking the value of those properties alone or combined into propositions (see Materials and Methods). Some properties that are useful for this analysis are: ERaACT, ERbACT, PKCeq1, and AbEQ2 (other properties also used have similar nomenclature). The properties ERaACT and ERbACT are true when ERalpha or ERbeta, respectively, are activated by estrogen. The properties PKCeq1 and AbEQ2 are true when PKCalpha is 1 or when Abeta is 2, respectively. The value of these (and other) properties alone or combined into propositions can be checked under specific sets of conditions. To determine why estrogen, in combination with NSAID and HIFblock, can bring Abeta to level 2 in the presence of CVD, the analysis takes place under conditions in which estrogen, NSAID, HIFblock, and CVD are all present.

In the Maude specification ALZHEIMERE, which specifies the estrogen-Aβ model, estrogen activates ERalpha and ERbeta, and it also brings PKCalpha to level 1. Because all three of these interactions are specified as rules, temporal-logic analysis can be used to explore the consequences of these interactions in all possible orders of occurrence. Some results of the temporal-logic analysis are shown in Table 3. To begin, temporal-logic analysis is used to check the value of the proposition ERaACT ⇒ AbEQ2 (where ⇒ is the logical connector “implies”). This proposition states that Abeta is at level 2 only if ERalpha is activated. This is false under the conditions of the analysis (Row 1 of Table 3), meaning that other actions of estrogen besides activation of ERalpha are necessary to keep Abeta at level 2.

**Table 3:** **Using temporal-logic to check the model in the presence of CVD when estrogen, NSAID, and HIFblock, but not caspBlock, are available**.

#	Proposition	Value
1	Activation of ERalpha implies Abeta equals two	False
2	Activation of ERalpha and ERbeta implies Abeta equals two	False
3	Activation of ERalpha, and PKCalpha equals one, imply Abeta equals two	False
4	Activation of ERbeta, and PKCalpha equals one, imply Abeta equals two	False
5	Activation of ERalpha and ERbeta, and PKCalpha equals one, imply Abeta equals two	True
6	Activation of ERalpha or ERbeta implies PKCalpha equals one	False
7	PKCalpha does not equal one until estrogen equals one	True
8	PKCalpha equals one implies Abeta equals two	False
9	Activation of ERalpha implies NEP equals three	False
10	Activation of ERalpha and ERbeta implies NEP equals three	True
11	NEP equals three implies Abeta equals two	False
12	PKCalpha equals one and NEP equals three imply Abeta equals two	True
13	Abeta does not equal two until PKCalpha equals one and NEP equals three	True

*Each row lists a proposition and its logical value (true or false). The row numbers appear in the leftmost column*.

The first five rows in Table 3 lists a set of propositions and their values that ends with the proposition (ERaACT ∧ ERbACT ∧ PKCeq1) ⇒ AbEQ2 (where ∧ is the logical connector “and”). This proposition states that Abeta is at level 2 only if ERalpha and ERbeta are activated and PKCalpha is at level 1. This statement is true but the previous propositions, whose antecedents are a conjunction (i.e., joined by “and”) of only two of the three properties in this antecedent, are all false (Rows 2 through 5 of Table 3). This means that Abeta will stay at level 2 only if estrogen activates ERalpha and ERbeta and brings PKCalpha to level 1 (i.e., all three of the functions of estrogen in the model are necessary).

Because PKCα activity is increased by estrogen directly (see Materials and Methods), it is useful to make sure that PKCalpha activity cannot be increased indirectly via ERalpha or ERbeta activation in the model. The proposition (ERaACT ∨ ERbACT) ⇒ PKCeq1 (where ∨ is the logical connector “or”) is false, meaning that neither ERaACT nor ERbACT activation is sufficient to raise the level of PKCalpha to 1 (Row 6 of Table 3). The proposition ∼ PKCeq1 U estEQ1 states that PKCalpha is not 1 until estrogen is present (estEQ1 is the property that estrogen is 1, and ∼ and U are the temporal-logic connectors “not” and “until,” respectively). This proposition is true, and the two propositions together prove estrogen raises the level of PKCalpha, but not via activation of ERalpha or ERbeta (Rows 6 and 7 of Table 3).

The temporal-logic analysis so far reveals an important difference between administration of estrogen and ERalphaAg: estrogen, but not ERalphaAg, raises the activity level of PKCalpha. Perhaps it is the increase in PKCalpha activity that allows estrogen to keep Abeta at the normative level 2 while ERalphaAg fails to do so? This question can be answered by checking the value of the proposition PKCeq1 ⇒ AbEQ2, which states that Abeta is 2 only if PKCalpha is 1. This proposition is false, meaning that PKCalpha at level 1 is not sufficient to keep Abeta at level 2 (Row 8 of Table 3).

Another difference between the effects of administration of estrogen and ERalphaAg concerns NEP. Expression of the enzyme NEP, which degrades Aβ, is upregulated by activation of ERα or ERβ, and upregulation by both receptors is additive (see Materials and Methods). In the model, estrogen, which activates both ERalpha and ERbeta, raises NEP to 3, and it is useful to check to ensure that ERalphaAg cannot raise NEP to 3 by itself. The proposition ERaACT ⇒ NEPeq3 (where NEPeq3is the property that NEP is at level 3) is false (Row 9 of Table 3). This means that activation of ERalpha alone is not sufficient to raise NEP to level 3. The proposition (ERaACT ∧ ERbACT) ⇒ NEPeq3 is true (Row 10 of Table 3), meaning that NEP reaches level 3 only if both ERalpha and ERbeta are activated. Thus, estrogen raises NEP to level 3 because it activates both ERalpha and ERbeta.

Perhaps it is this increase in NEP expression that allows estrogen to keep Abeta at the normative level 2 while ERalphaAg fails to do so? This question can be answered by checking the proposition NEPeq3 ⇒ AbEQ2, which states that Abeta is 2 only if NEP is 3. This proposition is false (Row 11 of Table 3), meaning that NEP at level 3 is not sufficient to keep Abeta at level 2. This leads to the question of whether the increases due to estrogen of PKCalpha and NEP are both necessary to keep Abeta at its normative level in the presence of CVD. This question can be answered by checking the values of two further propositions. The proposition (PKCeq1 ∧ NEPeq3) ⇒ AbEQ2 is true, meaning that Abeta stays at level 2 only if PKCalpha is 1 and NEP is 3. The proposition ∼ AbEQ2 U (PKCeq1 ∧ NEPeq3) is also true, meaning that Abeta is not 2 until PKCalpha is 1 and NEP is 3 (Rows 12 and 13 of Table 3). These two propositions together prove that Abeta is not at level 2 unless PKCalpha is at level 1 and NEP is at level 3. The temporal-logic analysis in this subsection suggests that, under conditions of low estrogen and incipient CVD, and with co-administration of an NSAID that promotes PPAR expression and a compound that blocks HIF, administration of estrogen itself would be more effective in lowering Aβ than administration of an ERα agonist.

## Discussion

The main finding of the simulations and analysis presented in Results is that, under conditions of very low estrogen and incipient CVD, the level of Aβ could be reduced, possibly to normative levels, with a combination of an NSAID that promotes PPAR expression, a compound that blocks HIF, and estrogen itself. The model suggests that estrogen would provide the main benefit, reducing Aβ directly (e.g., by enhancing NEP expression) and indirectly by reducing inflammation and OS (e.g., by enhancing SOD expression), thereby disrupting pathological processes that contribute to Aβ accumulation. With estrogen itself providing the main benefit, an NSAID and a HIF-blocker can each provide a small additional benefit, and these two benefits are additive in combination. The idea of combination estrogen/NSAID/HIF-blocker therapy for the treatment of AD in post-menopausal women is plausible.

The usage of estrogen and its derivatives for hormone replacement therapy is widespread but its benefits are still hotly debated (Barlow, 2008; Shifren and Schiff, 2010; Daniel, 2012; Marjoribanks et al., 2012; Moyer and On Behalf of the U.S. Preventive Services Task Force, 2012; Nelson et al., 2012). A consensus seeming to emerge is that estrogen replacement therapy may provide some neuroprotection if initiated soon after menopause.

The usage of estrogen is dwarfed by that of NSAIDs, which are among the most commonly used over-the-counter drugs. While epidemiological studies show that NSAIDs reduce AD risk, clinical trials testing NSAID efficacy in AD patients have not yielded positive results (Heneka et al., 2011). However, available evidence does not rule out the possibility that NSAIDs could be effective in combination with estrogen. NSAIDs potentially may reduce Aβ via several mechanisms including upregulation of PPAR expression (Sastre et al., 2006), and modulation of γ-secretase activity in such a way that APP processing by γ-secretase is reduced more than processing by γ-secretase of its other substrates (De Strooper et al., 2010). The model represents only the PPAR-upregulating function of NSAIDs and suggests that it may augment PPAR upregulation due to estrogen, thereby providing additional benefit. The γ-secretase-modulating role of NSAIDs could also provide some benefit, but that was not explored in this model.

Compounds exist that block HIF. One such is cilnidipine, which is also a calcium-channel blocker but it blocks HIF synthesis independently of its effects on ion channels (Oda et al., 2009). The discovery that cilnidipine blocks HIF synthesis raises the possibility that pharmaceuticals that specifically block HIF could be developed. The model suggests that such drugs would be useful for the prevention of AD, especially in combination with estrogen and NSAIDs. Compounds also exist that block the activation of caspase-3 (Dave et al., 2003), but the model suggests that blocking caspase-3 would be ineffective if co-administered with estrogen because estrogen already prevents caspase-3 activation directly, by increasing seladin-1 expression, and indirectly by reducing OS in the model.

The model also suggests that, in terms of reducing the level of Aβ, agonists of ERα would be substantially more effective than agonists of ERβ. Among the differential effects of ERα or ERβ activation, two are represented in the model: ERα activation increases seladin-1 expression but ERβ activation does not (Benvenuti et al., 2005), and ERα activation increases apoE expression while ERβ activation suppresses it (Wang et al., 2006). The three most common apoE isoforms are apoE2, apoE3, and apoE4, and the apoE4 isoform is associated with increased risk of AD (Saunders, 2000). These findings suggest that administration of an ERβ agonist could be the preferred treatment in carriers of the apoE4 allele (Wang et al., 2006). Otherwise, the model suggests that ERα should produce a dramatic reduction in Aβ by preventing it from triggering OS, which leads to further Aβ accumulation. The model also suggests that estrogen itself would be more effective than ERα agonists because estrogen, by activating both ERα and ERβ, brings NEP to a higher level than specific agonists of either receptor could do (Liang et al., 2010), and because estrogen directly activates PKCα (Alzamora et al., 2007), which in turn shifts APP processing away from the Aβ pathway by activating α-secretase (Cisse et al., 2011). The combined effects of estrogen alone prevent OS and also prevent the activation of cytokines.

In pulling many strands of data together, the model provides potential new insights into the contribution that estrogen makes to Aβ regulation. Its two main predictions are that, under conditions of low estrogen and incipient CVD, estrogen should reduce Aβ levels more than specific agonists either of ERα or ERβ, and NSAIDs and/or compounds that block HIF should provide additional benefit in combination with estrogen. However, any of the input/output configurations presented in Tables 1 and 2 could be taken as predictions of the model. These predictions could be tested experimentally using essentially the same *in vitro* and *in vivo* (mainly mouse) methods that were used to generate the data represented in the model. The results could be used to verify or correct the model, and verified predictions would provide avenues for development of new pharmacological strategies for the prevention and treatment of AD.

The model aggregates much of the current literature on Aβ regulation and the contribution of estrogen, but it is clear that the model could be expanded to include many more relevant interactions. Even still, at about 80 molecular interactions, the computational model could potentially generate a wide range of predictions, and far more than could be generated without it. The model demonstrates how declarative programming can be used to represent, simulate, and analyze large amounts of data on complex neurodegenerative processes and to generate experimentally testable predictions, and it stakes its claim as the starting point of a process in which model predictions are tested, the results are used to correct and extend the model, more predictions are generated and tested, and the process continues. The result is a model of ever increasing accuracy and explanatory power that provides ever more insight into the complex system of molecular interactions that underlie AD.

The model also illustrates the power of computation in representing and reasoning about complex neurodegenerative diseases, and clearly shows how it could be used to derive hypotheses concerning the potential benefits of multi-drug therapies. The approach is consistent with the general idea that complex problems require complex solutions, and that multifactorial disease processes such as neurodegenerative diseases should be combatted simultaneously on multiple fronts. Computational modeling of neurodegenerative disease processes using declarative programming provides a powerful methodology to aggregate data and search for potentially effective drug combinations. Support for expanding the model computationally and testing it experimentally should be provided. The current model is a call to arms.

## Conflict of Interest Statement

The author declares that the research was conducted in the absence of any commercial or financial relationships that could be construed as a potential conflict of interest.
